# The role of YAP in the control of the metastatic potential of oral cancer

**DOI:** 10.32604/or.2022.026085

**Published:** 2022-11-10

**Authors:** USAMA SHARIF AHMAD, KARTHIK SARAVANAN, HONG WAN

**Affiliations:** Centre for Oral Immunobiology and Regenerative Medicine, Institute of Dentistry, Barts and the London, School of Medicine and Dentistry, London, E1 2AT, UK

**Keywords:** Review, YAP, Phospho-YAP, Desmoglein-3, TAZ, Hippo pathway, desmosomes, OSCC, Oral cancer cell migration

## Abstract

The Yes-associated protein (YAP) is a downstream effector of the Hippo pathway and acts as a key transcription co-factor to regulate cell migration, proliferation, and survival. The Hippo pathway is evolutionarily conserved and controls tissue growth and organ size. Dysregulation and heterogeneity of this pathway are found in cancers, including oral squamous cell carcinoma (OSCC), leading to overexpression of YAP and its regulated proliferation machinery. The activity of YAP is associated with its nuclear expression and is negatively regulated by the Hippo kinase-mediated phosphorylation resulting in an induction of its cytoplasmic translocation. This review focuses on the role of YAP in OSCC in the context of cancer metastatic potential and highlights the latest findings about the heterogeneity of YAP expression and its nuclear transcription activity in oral cancer cell lines. The review also discusses the potential target of YAP in oral cancer therapy and the recent finding of the unprecedented role of the desmosomal cadherin desmoglein-3 (DSG3) in regulating Hippo-YAP signaling.

## Introduction

YAP is a nuclear transcription cofactor and a key downstream effector of the evolutionarily conserved Hippo pathway which governs cell differentiation, tissue growth and organ size. The pivotal role of YAP is to regulate cell proliferation and amplify the tissue-specific progenitor cells essential for tissue renewal and regeneration and therefore to govern organ size regulation. Thus, it is not surprising that upregulation of YAP is found in various cancers, including oral squamous cell carcinoma (OSCC), where YAP can incite tumor initiation, progression and metastasis. YAP amplification is found to be associated with poor survival in patients with head and neck cancers, as well as high-grade OSCC, suggesting that inhibition of YAP or its downregulation could improve the prognosis for some patients with oral cancer. In addition, increasing evidence has suggested that nuclear accumulation of YAP is related to treatment resistance, including common chemotherapeutic agents such as Cisplatin and Cetuximab used in OSCC, as well as resistance to radiotherapy [1–3]. Therefore, targeting YAP could represent a key opportunity in mitigating tumor progression and metastasis and in improving disease-free survival. This review highlights the paramount role of YAP in OSCC metastatic potential with our recent extended work in other systems. The discussion also covers the recent findings on the heterogeneity of the Hippo-YAP pathway in oral cancer cell lines and provides a new paradigm of cell migration control.

## Oral Cancer

Oral cancer is traditionally defined as malignant lesions that arise on the lips or within the oral cavity [4]. Oral cancers are among the ten most common types of cancers worldwide and account for 2%–4% of all cancer cases globally [5]. The most common type of oral cancer is squamous cell carcinoma derived from stratified squamous epithelium in the oral mucosal membrane and accounts for an estimated 80%–95% of all oral neoplasms [6,7]. Global reporting of cancers tends to group cancers of the oral cavity, pharynx, and larynx as head and neck cancer, representing the sixth most common form of cancer worldwide [8]. Oral cancer including those derived from salivary glands is diagnosed in around 300,000 patients and is associated with 145,000 deaths per year [9]. However, there is marked variation in the incidence of oral cancers across the globe, reflecting the prevalence of risk factors associated with the condition.

The most common risk factors associated with the development of OSCC are the use of tobacco and excessive alcohol consumption [10]. It is hypothesized that alcohol increases the permeability of the oral epithelium making it more susceptible to carcinogens present in tobacco [10]. Oral cancer is more common in men (2~3-fold) than in women, which may broadly reflect differences in alcohol consumption and smoking behaviors between the sexes [11]. It is currently believed that HPV plays a ‘hit and run’ role in causing carcinogenesis, however; others have suggested that HPV infection is not enough to cause malignant transformation of oral mucosal cells *per se* unless cells are exposed to carcinogens [12,13]. Studies have shown that HPV contributes to carcinogenesis by two virus-encoded proteins. The first is the E6 protein, which promotes the degradation of the tumor suppressor protein, p53. The other is E7, which promotes degradation of the retinoblastoma protein, which is responsible for preventing cell cycle progression from the G1 to S phase. Other risk factors include exposure to ultraviolet (UV) radiation, poor oral hygiene, and dietary/vitamin deficiencies [14–16]. Early diagnosis and treatment of OSCC are imperative to enhance patient survival and reduce the need for extensive surgery [17,18]. Unfortunately, 60%–80% of OSCC cases present at advanced stages. In general, the malignant process begins from initial cellular dysplasia to carcinoma *in situ*, which progresses to malignant changes, and then metastatic spread. The first clinical signs of OSCC can be dysplastic changes in the mucosal membrane, including erythroplakia and leukoplakia, the two most common conditions [7]. Leukoplakia applies to a white patch on the oral mucosa [19]. Histological examination shows marked hyperplasia of cells, hyperkeratosis, and acanthosis, with dysplastic and non-dysplastic lesions [18]. Leukoplakia is strongly associated with tobacco use and alcohol consumption and has a worldwide prevalence of approximately 2% [20]. Around 1% of leukoplakia cases undergo malignant transformation per annum [9,21]. Erythroplakia is described as a red, often velvety, patch in the oral mucosa, which is not associated clinically or pathologically with any other condition [18]. These lesions are often more strongly linked with the potential for malignant transformation than leukoplakia. Indeed, both erythroplakia and leukoplakia are strongly associated with dysplastic changes and carcinoma *in situ* [9].

The treatment of OSCC includes chemotherapy, radiotherapy, surgical interventions, as well as inhibitors for epidermal growth factor receptor (EGFR), cyclo-oxygenase-2 (COX-2) and other therapeutic agents targeting aspects of molecular pathways associated with malignancy [22]. An example of these pathways which has attracted increased attention in recent years is the Hippo pathway. Treatments directed towards crucial molecular markers of tumors, including mutations associated with tumor growth and survival, have been an area of interest in general oncology. Targeting critical molecules involved in specific signaling pathways related to cancer cell proliferation, division, invasion, and metastasis has been shown to improve patient survival with low toxicity levels in patients [23]. Despite the value of traditional treatment options, the 5-year survival rate following diagnosis is only 50%, and this rate falls to 30%–40% in patients at advanced stages [24]. Targeted therapy has the advantage of avoiding damage to healthy tissue, which is commonly seen with surgery, chemotherapy, or radiotherapy [5]. While drugs targeting general molecular pathways involved in OSCC may be a good approach, there is a need to consider specific efficacy and the characteristics of the molecular milieu associated with OSCC to guide treatment options. The Hippo pathway draws increased interest because it involves in regenerative and pro-cancerous functions. Hence, the need to expand therapeutic targets and to study the molecular pathways and processes associated with OSCC metastasis are invaluable in aiding future treatment options [25].

## The Hippo Pathway

The Hippo signaling pathway was first discovered during the 1990s in *Drosophila melanogaster* as a result of screening for tumor suppressor genes and genes associated with organogenesis and development [26,27]. Key components of this pathway were identified during these genetic screens. Inactivation of several components in this pathway in *D. melanogaster* leads to the same phenotype where tissue overgrowth was pronounced [28]. Thus, the Hippo pathway was first noted to be an essential regulator of organ size, development, and homeostasis in the organism [29,30]. There are over 30 components identified in the Hippo pathway that comprises upstream regulatory factors, kinase core, downstream effectors, and transcription factors, which suggest a complex signaling cascade of this pathway. In principle, this pathway consists of two modules, one of which contains the cytoplasmic serine/threonine kinase cascade that is activated by diverse upstream cues and are considered tumor suppressors. The other module is the nuclear transcription components that are the downstream effectors of Hippo signaling, which act as oncogenes [31]. The key components of the Hippo pathway such as MST1/2, LATS1/2, SAV1, MOB1A/B, the orthologues of *Hippo, Warts, Salvador, and Mats* in *D. melanogaster* respectively, share similar functions and regulate aspects of cell growth and tissue size control [32–35].

**Figure 1:**
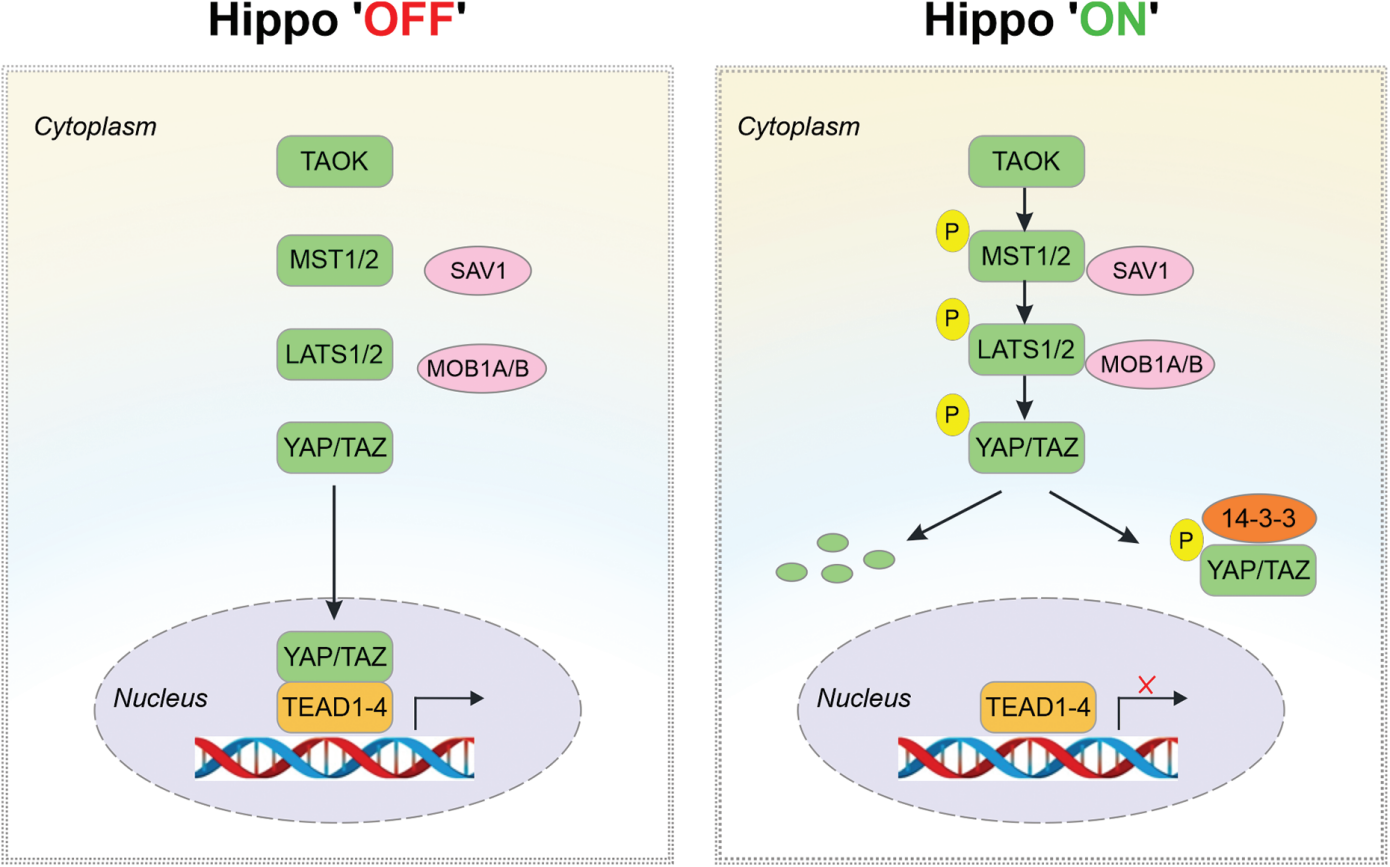
The Hippo pathway in mammals. When the pathway is inactive (Hippo ‘OFF’), YAP/TAZ remain unphosphorylated and are localized to the nucleus, where they bind to TEAD and activate gene transcription. When the pathway is active (Hippo ‘ON’), upstream stimuli phosphorylate and activate TAOK kinases which phosphorylate MST1/2. MST1/2 then phosphorylates LATS1/2, which is facilitated by adaptor proteins SAV1 and MOB1A/B. Finally, the activated LATS1/2 phosphorylates YAP/TAZ, leading to YAP/TAZ ubiquitination and proteolytic degradation or allowing their interaction with protein 14-3-3 and cytoplasmic retention, rendering them unable to maintain their action in the nucleus. TEAD refers to the transcriptional enhanced associate domain, and TAOK refers to serine/threonine protein kinases.

The functions of the Hippo pathway are diverse and reflect cellular regulatory events that control tissue growth and development. Primarily, the pathway serves to promote cell death and differentiation while inhibiting cellular proliferation across different species [36]. The study of the key components in the Hippo pathway has advanced the knowledge of the mechanisms involved in cell growth control and organogenesis while highlighting the putative roles of individual proteins in this pathway (Fig. 1). Phosphorylation of MST1 at Thr183 and MST2 at Thr180 triggers MST1/2 activation [37]. MST1/2 can also undergo auto-activation through MST1/2 dimerization and autophosphorylation [38]. The active MST1/2 then phosphorylates the adaptor proteins Salvador family WW domain-containing protein 1 (SAV1) and MOB kinase activator 1A/B (MOB1A) [39]. Both scaffold proteins play a pivotal role in sequestering and facilitating interactions between MST1/2 and LATS1/2. MOB1A/B forms a complex with LATS1/2 allowing MST1/2 to phosphorylate LATS1 at Thr1079 and LATS2 at Thr1041, which subsequently leads to LATS1/2 activation. In addition, a cascade of autophosphorylation events also occurs within an activation loop [40,41]. Once LATS1/2 is activated, it can directly phosphorylate Yes-associated protein (YAP) and transcriptional co-activator with PDZ-binding motif (TAZ), leading to their cytoplasmic translocation and inactivation [42]. The loss of the core components (MST1/2, SAV1, MOB1A/B and LATS1/2) results in an opposite effect with the upregulation of YAP/TAZ and their mediated target gene expression via binding to TEAD family transcription factors and inducing cell proliferation [43,44]. Thereby, uncontrolled growth is seen following the loss of Hippo inhibitory effects on YAP/TAZ [45]. Similar observations of uncontrolled tissue growth are also made in mice by gene deletion studies for proteins that inhibit YAP/TAZ activity [46]. Therefore, the regulation of the Hippo pathway, including the YAP/TAZ proteins, appears to have significance in the context of development, particularly in diseases such as cancer, where poorly regulated cell growth is a hallmark of the condition.

## Yes-Associated Protein (YAP)

### Molecular structure of YAP

YAP was first identified in mammals as the homologous protein to *D. melanogaster* Yki in 1994 and the first protein to be discovered with a WW domain [47]. YAP has a paralogue in mammals, a gene that has evolved within the same species due to duplication termed TAZ (transcriptional co-activator with PDZ-binding motif). The discovery of TAZ was found later in the year 2000 [48]. The discovery of both YAP and TAZ has led to a detailed understanding of the molecular structure of these essential regulators of embryonic development and regulators of cell growth and proliferation in adult cells. It is important to note that TAZ which is encoded by the *WWTR1* gene, can be easily confused with another protein called Tafazzin. However, the Tafazzin protein is entirely unrelated to the Hippo pathway and is encoded by the *TAZ* gene [49,50].

**Figure 2:**
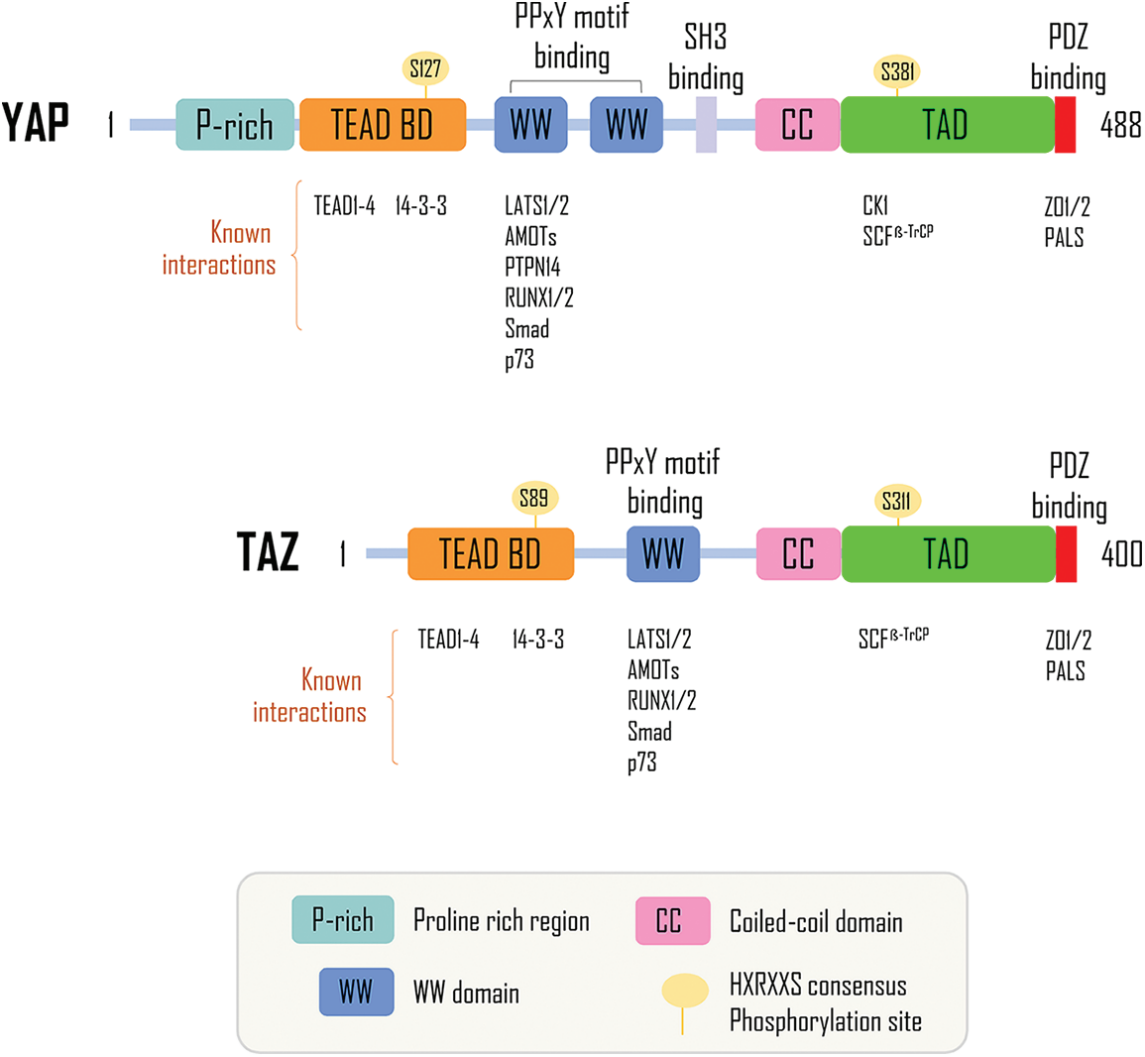
Regulatory domains of YAP/TAZ proteins. YAP comprises an N-terminal proline-rich region (P-rich), followed by a transcription factor interacting domain (TEAD), two WW domains, a Src homology 3 binding motif (SH3), a coiled-coiled motif (CC), and a transcriptional activation domain (TAD) and finally, the C-terminal region comprises a PDZ-binding motif. TAZ has a similar structure as YAP; however, it does not have a proline-rich region, a second WW domain, and an SH3-binding motif. The proteins known to interact with different domains are shown below. TAZ has a 44% identity to YAP [25]. Adapted from [57].

The gene encoding YAP is located on chromosome 11q22 in humans and is ubiquitously expressed during development, indicative of widespread roles in developmental regulation [51]. YAP exists as two main subtypes, *YAP1* and *YAP2*, but little evidence is obtained for differential effects of these subtypes within the Hippo pathway [52]. The molecular structure of YAP comprises an N-terminal region rich in proline and a C-terminal PDZ binding motif [53] and between them, there are specific amino acid sequences consistent with transcriptional enhanced associate domain (TEAD) binding sites, WW regions (two tryptophan residues), an SH3-binding motif, a coiled-coiled domain and transcriptional activation domain (TAD) [25] (Fig. 2). TAZ has similar molecular structural and functional domains, although it lacks a second WW domain, SH3-binding motif, and a proline-rich region at the N-terminal [36]. The significance of some of these structural features has been considered in the literature. For instance, the WW binding domain is thought to recognize proline-tyrosine motifs, which may regulate the translocation and activation status of YAP and TAZ [51]. The PDZ domain of the C-terminal is common to many proteins and may have functional significance for YAP in facilitating interaction with many potential protein effectors and regulators [36]. LATS1/2, AMOT, and PTPN14 can directly interact with YAP through their PPxY motifs and the WW domains of YAP [54,55]. In contrast, specific proteins are dependent on the phosphorylation status of YAP for interaction, such as protein 14-3-3 and α-Catenin [56]. The domain architecture of both YAP and TAZ are indicative of interactions with proteins and transcription factors, including the TEAD family and factors with proline-tyrosine motifs [25]. However, it has been suggested that despite their similarities, YAP and TAZ can regulate different sets of genes in a cell context-dependent manner. Therefore, the regulation and function of YAP should be considered carefully to provide a basis for examining the role of this protein, as a specific member of the Hippo pathway.

### Regulation and biological functions of YAP

YAP is a tightly regulated protein, which is influenced by multiple intracellular and extracellular stimuli facilitated through components of the Hippo pathway. Therefore, phosphorylation of YAP (or TAZ) represents a critical event that determines the location of the protein within the cell, as well as the activity and stability of the protein [25]. As both YAP and TAZ have similar functions in the Hippo signaling pathway, the following sections will focus on YAP, but the findings can be considered applicable to both proteins unless evidence for divergence in function or regulation is described.

One of the key features of YAP activity is the localization of the protein within the cell [16]. Nuclear localization is crucial for YAP as a transcriptional activator and subsequent promotion of cell growth and proliferation [36]. The regulation of YAP is primarily based on its phosphorylation status and localization characteristics of the protein. The phosphorylation of YAP (e.g., by LATS1/2) leads to YAP cytoplasmic retention while binding to protein 14-3-3. In addition, LATS1/2-induced phosphorylation can lead to ubiquitination-mediated proteasomal or lysosomal degradation of YAP, all of which prevent YAP from direct interaction with nuclear transcription factors [42,58,59]. YAP has five substrate consensus sequence HXRXXS/T motifs that are recognized and phosphorylated by LATS1/2: serine-61, serine-109, serine-127, serine-164, and serine-381. On the other hand, TAZ has four HXRXXS/T motifs, which LATS1/2 phosphorylates: serine-66, serine-89, serine-117, and serine-311 [60,61]. However, the well-studied phosphorylation sites are serine-127 (S127) which results in 14–3–3 binding and cytoplasmic sequestration of YAP, and serine-381 which primes YAP for ubiquitination. For TAZ, the most relevant residues are serine-89 and serine-311 [62]. On the contrary, phosphorylation of YAP at S127 may not necessarily be an inactivating event, as YAP-S127 is found in the nucleus in keratinocytes [63–67], although this warrants further experimental evidence and explanation.

YAP is a transcriptional co-activator and the primary nuclear effector of the Hippo pathway. YAP does not bind directly to DNA due to the absence of DNA-binding domains. When unphosphorylated, YAP translocates to the nucleus, where it interacts with DNA-binding transcription factors to regulate gene expression. Interaction with TEAD1-4 transcription factors is YAP's primary mechanism of action in promoting specific biological functions [53]. TEADs are sequence-specific transcription factors, and the formation of the YAP-TEAD protein complex mediates the main transcriptional output of the Hippo pathway with the regulation of genes related to cell growth, proliferation, migration, apoptosis, and other homeostatic mechanisms associated with survival [45,68,69]. The physical interaction of TEAD with YAP is mediated by the C-terminal region of TEAD with the N-terminal region of YAP [53,70]. Similarly, YAP has been shown to interact with several other transcription factors such as RUNX3, TBX5, p73, and ERBB4, leading to a wide range of biological functions [71–74].

When YAP is phosphorylated, TEADs bind to the vestigial like family member 4 (VGLL4) transcriptional cofactor in the nucleus and function as transcriptional repressors. The interaction of YAP with TEADs dissociates VGLL4 from TEADs and allows activation of TEAD-mediated gene transcription [75]. YAP activity is directly associated with the regulation of a range of target genes such as cysteine-rich angiogenic inducer 61 (*CYR61*), connective tissue growth factor (*CTGF*), *MYC*, and Vimentin (*VIM*) [76–79]. Other genes include mutant *TP53, NEGR1* [53] and *UCA1*, which are associated with tumor progression and maintenance of tumor activity [80]. Under physiological conditions, growth signaling is tightly regulated, but in the context of cancer, dysregulation of the activity of these genes may facilitate tumor growth and metastasis [36]. Accordingly, YAP plays a vital oncogenic role. Interestingly, YAP can have either oncogenic or tumor suppressive functions depending on which DNA-binding transcription factors it binds to, which in turn is determined by cellular events. For example, Jang et al. showed that the phosphorylation of YAP by LATS1/2 switches the interaction of YAP-TEAD4 to YAP-RUNX3 (RUNXs are master regulators of development). In this context, at low cell density, YAP binds to TEAD4 to promote growth, but at high cell density, upon phosphorylation, the YAP-TEAD4 complex disassociates enabling interaction with RUNX3 instead [81]. Thus, events such as cell compactness can govern the interaction of YAP with different DNA-binding partners determined through its phosphorylation status and cell context [81]. Another study demonstrated that YAP interacts with p73, akin to p53, in that p73 can induce cell cycle arrest and apoptosis [73]. In summary, the biological functions of YAP are tightly regulated in a cell context and cell density-dependent manner and appear to be broadly consistent with those attributed to Hippo signaling, with YAP serving as a vital component of this pathway.

### YAP is over-expressed in oral cancer

Chronic exposure to risk factors such as tobacco and alcohol in the normal oral mucosa leads to genetic alterations of several pathways such as p53, p16, cyclin D1, and the retinoblastoma protein resulting in uncontrollable cellular proliferation and rendering the inability of cells to respond to stress or DNA damage, together contributing to tumorigenesis [82,83]. Currently, the relationship between aetiological factors of oral cancer and the Hippo-YAP pathway is limited at present. However, given the vast array of genetic alternations found in oral cancer, it is highly likely YAP might not play a role in initiating the oncogenic process (i.e., consequent to oncogenesis, rather than causative), and that metastasis is YAP-driven in OSCC. Thus YAP could be an important therapeutic target based on several *in vitro* and *in vivo* studies [1,84–87]. YAP is known as a key player in cell migration via suppression of E-cadherin-mediated adherens junctions and enhancement of EMT [88–91]. YAP is also considered a biomarker for metastasis and resistance to EGFR inhibitors (Gefitinib & Cetuximab) in HNSCC [1,87].

The oncogenic role of YAP has been explored in multiple forms of carcinomas, affecting a variety of cell lines and tissue types. The YAP gene is found to be significantly amplified and overexpressed in OSCC, which is an important indicator of the potential function of this gene in oncogenesis. It is noted that YAP expression is elevated, and the protein is localised to the nucleus promoting cell proliferation, invasiveness, and survival of many forms of SCCs, including cutaneous, oesophageal, head and neck, oral, and cervical SCCs [55,92–95]. A study by Chen et al. showed that the expression level of YAP was elevated more in OSCC tissues than in adjacent normal tissues [96]. Another study by Szelachowska et al. examined 127 patient tissue samples that underwent surgery alongside adjuvant radiation therapy. Using immunohistochemistry, they found significantly increased YAP expression in cancer-associated fibroblast cells, leading to a poor prognosis in these patients [97]. In cutaneous SCC, YAP acts as a downstream molecule of protein 14-3-3, accumulates in the nucleus, and inhibits the differentiation of skin cells, leading to tumour formation [95,98]. Similarly, in oesophageal SCC, clinical observations showed that YAP activity is high, localised to the nucleus, and is associated with a poor prognosis due to immune evasion and aggressive tumour growth [94]. In head and neck SCC, YAP expression was significantly high in tumours [93]. Another study found that YAP activity is increased due to ACTL6A/ P63 complex activity on WWC1, which promotes tumorigenesis and reduces the prognosis of the condition [99]. Finally, in cervical SCC, Liu et al. reported that YAP expression was significantly increased in cervical SCC compared to normal tissue as well as correlated with tumour differentiation, metastasis, and early recurrences [55]. Furthermore, *in vivo* studies show that conditional deletion of YAP in mice in basal cell carcinoma can prevent tumour formation, while YAP activity is associated with many regulatory events suggestive of uncontrolled growth as well as immune evasion and metastasis of tumour tissues [100]. In mouse models of SCCs, YAP is overexpressed and has a functional role in tumour formation and progression. Nevertheless, although the association between YAP and oncogenic processes is apparent in many tumour types, these associations are often seen in conjunction with other gene mutations and protein dysfunctions. Consequently, the clinical significance of YAP (or YAP/TAZ) in many types of cancers is uncertain [25]. Therefore, while it is essential to recognise that high levels of YAP have been seen in association with OSCC, this alone does not indicate the validity of YAP as a target for OSCC therapy.

A wide range of molecular and genetic heterogeneity is found in primary tumours. This heterogeneous nature often influences the clinical outcome of the disease and helps select and predict response to therapy. *In vitro* studies based on the panels of cancer cell lines can help identify the heterogeneity present in primary tumours and these cell lines can be used as tools for understanding the pathogenic processes of clinical disease, such as metastasis [101–103]. For instance, our recent study of the Hippo-YAP pathway in a panel of authentic OSCC cell lines derived from mouth floor and buccal mucosa showed overexpression of *YAP1*, as well as *WWTR1* [86], the results that are consistent with other reports [31,104,105]. Three groups in our gene signature study (including 46 genes) included: (1) the Hippo pathway (21 genes); (2) cancer biomarkers (8 genes); and (3) intercellular anchoring junction-associated genes (17 genes) [86]. The first group of genes consisted of *YAP1/WWTR1*, Hippo pathway components, and transcription factors that were all found to be upregulated in OSCC cell lines. A negative feedback loop likely causes the increased expression of upstream Hippo kinases upon YAP activation [106]. *LATS1/2* have been reported as the direct target genes of YAP/TAZ [107]. YAP/TAZ activation and concomitant enhancement of TEAD4 were also identified that have been implicated in the progression of HNSCC [31]. The second group of cancer biomarkers showed upregulation in carcinoma cell lines compared to normal cell lines [107]. The strong cancer signature with high levels of cancer biomarker expression in OSCC cells fits the general paradigm of cancer and indicates the authentic nature of the resources of cancer cell lines. The last group included cell junction-associated genes of adherens junctions and desmosomes, with the former being known as negative regulators of YAP. In general, there appeared a tendency of reduction in these cell junctional genes in dysplasia and cancer cells, whereas huge variations were observed in OSCC lines [86]. Overall, our findings have indicated complexity and variations of the Hippo-YAP pathway in OSCC cell lines which agree with another report [104] and demonstrate a general trend of elevated YAP expression in OSCC cells.

### YAP is required for efficient oral cancer cell migration

Chronic exposure to risk factors in the normal oral mucosa leads to alterations of several pathways resulting in uncontrollable cell proliferation and tumorigenesis [82,83]. YAP is overexpressed in OSCC but the expression and amplification profiles may represent a secondary effect of tumour growth. Due to the vast array of genetic alternations as well as the aberrant effects of tumour activity [108], it was reasoned that YAP might not play a role in driving the oncogenic process. Therefore, we hypothesised that YAP may drive the metastatic potential of OSCC cells. Thus, we analysed two OSCC model systems (buccal carcinoma lines H157 and H413) that exhibited different levels of YAP expression. The H157 cell line had the highest expression levels of YAP among the cancer lines in the study, whereas the H413 cell line exhibited moderate levels of YAP. Using the loss-of-function study approach (transient YAP siRNA transfection with two hits), we demonstrated that YAP depletion in both cell lines significantly suppressed the capacity of collective cell migration, and this effect was also seen in cells with the treatment of mitomycin C (MMC) that inhibits DNA replication and arrests the growth of cells [109], compared to the respective controls [86]. Furthermore, time-lapse microscopy to track random cell migration of small colonies also showed consistently an inhibition of cell migration in YAP siRNA-treated cells. In line with these findings, YAP is reported as a key driver in cell migration via suppression of E-cadherin-mediated adherens junctions and enhancement of EMT [85,88–91]. Moreover, YAP is considered a biomarker for metastasis and resistance to EGFR inhibitors (Gefitinib & Cetuximab) in HNSCC [1,110]. Hence, these findings suggest that YAP is required for efficient OSCC cell migration and is thus a promising drug target to halt cancer progression and metastasis that account for the vast majority of cancer-related deaths [111,112].

Using RT-qPCR, we showed that YAP knockdown in H157 and H413 significantly reduced *YAP1* target genes, cell cycle and survival-associated genes [86]. However, the Western blotting results revealed no evident change in cell cycle proteins in YAP knockdown cells in both lines compared to controls, indicating the primary function of YAP in OSCC is to control cell motility, at least in the cell lines we studied. Protein analysis of adherens junctions and desmosome proteins showed upregulation of E-cadherin, α-Catenin, PKP1, and PKP3 (plakophilins 1.3) in both cell lines with YAP knockdown, in particular, DSG3 that showed a signification induction coupled with enhanced membrane localisation in response to YAP silencing in both cell lines [86]. A similar finding was also found in our earlier report [113], suggesting a negative regulation between DSG3 and YAP. These findings shed new light on the desmosomal proteins such as DSG3 which serve as the upstream regulators of the Hippo-YAP pathway, in addition to adherens junction proteins [56,67,112,113] (discussed below). Taken together, our studies suggest that YAP is required for efficient oral cancer cell migration, with YAP depletion resulting in significant inhibition of cell migratory capacity in both the collective and colony settings. Moreover, YAP deficiency affects the transcription program of cell proliferation but to a lesser degree on the protein expression.

### Heterogeneity of YAP and TAZ in OSCC cell lines

Although YAP knockdown inhibited cell migration, interestingly, our close inspection identified different migration patterns between the two OSCC cell lines. H413 cells appeared to migrate significantly faster than H157 cells and this difference was even more revealing when compared to the cells with YAP knockdown [86]. In contrast to H157 which showed almost complete abrogation of cell migration by YAP knockdown, H413 cells had only partial inhibition of cell migration, suggesting different phenotypes of YAP dependency, i.e., a YAP-dependent trait in H157 and a mixed phenotype of YAP-dependent and independent trait in H413. To scrutinise the different phenotypes, we performed the following studies: first, we analysed the expression of focal adhesion kinase (FAK) and phospho-FAK (p-FAK) in both lines. FAK is a cytoplasmic protein-tyrosine kinase located at the sites of integrin-mediated focal adhesion and plays a vital role in regulating the cell migration-associated signalling in tumour cells. Increased FAK and its phosphorylation occur in many cancers, in particular in advanced-stage solid tumours [114,115]. Western blotting analysis indicated higher expression levels of FAK and p-FAK in H413 than in H157 cells. In addition, confocal image analysis of FAK- and p-FAK-associated focal adhesion revealed enhanced streak-like focal adhesions located at the edges of colonies in H413 cells but largely restricted at the cell periphery in H157 with a smaller size and a reduction of both protein expression. Hence, the residual migratory activity of H413 cells might be attributed to augmented FAK-mediated focal adhesion activity. Next, a direct comparison of the heat map in these two lines observed their strikingly distinct gene signatures [86]. H157 displayed elevated *YAP1* expression, however, several *YAP1* target genes and *WWTR1* were downregulated in this line, indicating low YAP/TAZ activity. In sharp contrast, the H413 line showed a low YAP expression but an elevated expression of *YAP1* target genes and *WWTR1*, suggesting high YAP/TAZ activity in this line, the results agreed with the YAP/TAZ luciferase assay (with a 5-fold increase). In line with this finding, genes related to cell-cell junctions were significantly downregulated in H413 compared to H157, further supporting the notion of high YAP/TAZ activity in H413 since the loss of junctional components leads to nuclear accumulation and activation of YAP [112].

Many studies focus only on the specific role of YAP or TAZ without considering the interplay of both proteins [116]. A recent finding by Chai et al. reported that only a subset of cancer lines in which the loss of YAP can be compensated for by its paralog TAZ [104], suggesting a partial overlap of YAP and TAZ functionalities. To verify whether YAP nuclear localisation is associated with its activity, we performed confocal microscopy for YAP staining in the two cell lines and observed predominant YAP nuclear localisation in H413 but cytoplasmic distribution in H157. Hence our study provides direct evidence that YAP nuclear localisation is correlated with its transcriptional activity, rather than its abundance in *YAP1* and protein expression, and enhanced YAP activity is associated with reduced expression of cell junctional components.

In addition, the effect of YAP knockdown on TAZ expression and localisation was analysed. We showed that YAP knockdown caused no marked reduction in *WWTR1* and TAZ protein expression. Immunofluorescence analysis of TAZ showed prominent TAZ nuclear staining in response to YAP knockdown in H413, suggesting active TAZ in this line. These findings led us to hypothesise that the YAP-independent phenotype of H413 cells might be associated with TAZ and FAK activity that had an impact on cell migratory capacity [117]. To address this question, we performed a single or double knockdown of YAP and TAZ in H413 cells before monitoring the ability of cell migration. We showed that only double YAP/TAZ knockdown resulted in substantial inhibition of cell migration compared to YAP or TAZ knockdown alone, suggesting an additive effect of YAP and TAZ functions on cell migration in H413 cells and that the residual migratory property in H413 YAP knockdown cells was attributed to the action of TAZ, the result confirmed by the YAP/TAZ luciferase assay. Collectively, these findings suggest that YAP and TAZ have non-overlapping activities in controlling OSCC cell migration and also indicate the heterogeneity of the Hippo-YAP/TAZ pathway in OSCC cells that has an impact on cell migratory behaviour *in vitro* and metastatic potential *in vivo*.

To address whether the increased TAZ activity was responsible for the elevated FAK/p-FAK expression in H413 cells, we analysed the expression of both proteins in cells with TAZ knockdown alongside YAP knockdown and double YAP/TAZ knockdown. However, there was no evident difference in FAK/p-FAK expression between TAZ knockdown and control cells, indicating that FAK/p-FAK may not contribute to the TAZ-mediated residual migratory activity of H413 YAP knockdown cells. On the other hand, YAP knockdown or YAP/TAZ double knockdown resulted in a reduction of p-FAK, suggesting that YAP is responsible for regulating FAK-mediated focal adhesions in H413 cells, the result agreed with other reports [118,119]. Thus, TAZ may regulate a different set of genes (e.g., ECM) associated with cell motility and its enhanced activity is responsible for the residual migratory activity in H413 cells. Collectively, our findings support the notion that both YAP and TAZ are not identical twins and have distinct transcriptional programs that govern cancer cell metastatic potential [116,117]. Therefore targeting YAP alone may not be effective in cancer treatment.

In summary, our study demonstrated distinct phenotypes in terms of YAP dependency. While H157 displayed a YAP-dependent trait, H413 manifested a mixed phenotype of YAP-dependent and independent features, with the latter attributed to TAZ activity. This finding underscores the clonal complexity and heterogeneity of the Hippo-YAP pathway in oral cancer cells, suggesting that a higher level of diversity in this pathway could exist in OSCC as well as in other cancers.

### YAP as a promising target for oral cancer treatment

The amplification of YAP is associated with poor survival in patients with head and neck cancers and high-grade OSCC, suggesting inhibition of YAP or its downregulation could improve the prognosis for some patients with oral cancer [93,94]. In addition, nuclear accumulation of YAP is related to treatment resistance, including common chemotherapeutic agents, such as Cisplatin and Cetuximab, used in OSCC, as well as resistance to radiotherapy [1–3]. The potential for YAP to serve as a therapeutic target in oral cancer has drawn increasing interest recently, reflecting important observations about the expression and the putative oncogenic functions of YAP in cancers [120]. Targeting YAP could represent a key opportunity in mitigating tumour progression and metastasis and in improving the disease-free survival of patients with oral cancer. Experimental evidence on inhibitor therapy that directly or indirectly targets YAP suggests some efficacy of this approach in managing SCCs [25]. However, molecules that can inhibit YAP or prevent/reduce its accumulation or overexpression are limited, with few agents being reported in the literature. Drugs or small molecules that target YAP/TAZ can be divided into those that affect the nuclear localisation of YAP/TAZ and those that compete with YAP/TAZ in binding to transcription factors. Agents that decrease YAP nuclear localisation either by activating its phosphorylation or promoting its nuclear-cytoplasmic shuttling include A35 and dichloroacetate [121,122]. In addition, Oku et al. found three drugs Pazopanib, Statins, and Dasatinib that inhibit the nuclear localisation of YAP and TAZ by inducing YAP/TAZ phosphorylation in breast cancer cells [123]. Competitive inhibitors of YAP include VGLL4, BRD4, and CA3 [124–126]. When YAP is phosphorylated, TEADs bind to the VGLL4 transcriptional cofactor in the nucleus and function as transcriptional repressors. A peptide mimicking VGLL4 was shown to inhibit the growth of gastric cancer [124]. Furthermore, the small molecule *CA3* was shown to suppress YAP expression and inhibit cell migration in OSCC cell lines [127].

Another YAP inhibitor is Verteporfin (VP), which has been approved by the Federal Drug Administration used in photodynamic therapy for neovascular macular degeneration and has been shown to have some efficacy in the management of SCCs [24]. Early studies reported that VP was associated with the disruption of the YAP-TEAD complex [128]. A growing body of evidence has identified that VP possesses anti-tumoural activity even in the absence of photo-activation [129]. Various studies have found that VP can reduce the proliferation, and metastatic potential, lower chemotherapy resistance and increase sensitivity to radiotherapy of various human cancers, including OSCC [130–132]. A study by Brodowska et al. found that, without light activation, VP inhibited proliferation, migration, and angiogenesis of human retinoblastoma cell growth *in vitro*, in a dose-dependent manner via downregulation of the YAP-TEAD complex and target genes (*CYR61, CTGF* and *CMYC*) [133]. A similar finding was reported in head and neck SCC cell lines [24]. The photodynamic effects of the drug were not necessary to promote anti-oncogenic activity and YAP was a key to the drug’s inhibitory mechanism. Furthermore, genes related to epithelial-to-mesenchymal transition (*YAP1, Snail, EGFR and CTNNB1*) were found to have a lower expression level than untreated cell lines. In contrast, E-cadherin expression was increased (suggesting a reduction in the migratory potential of cells), while expression of genes associated with metastatic potential was reduced [24]. Another report showed that knockdown of YAP or VP treatment in CAL27 cells inhibited cell proliferation while augmenting apoptosis and led to a decrease in *BCL2* and *CMYC* genes. These findings were replicable *in vivo* by using a tumour xenograft model. Therefore, YAP is associated with signalling events that promote tumour growth and high-grade tumour activity, including resistance to cell death signalling [96]. Another study conducted by Omori et al [134] generated mouse models with tongue-specific deletion of MOB1A/B. These mice had hyper-activation of endogenous YAP, leading to invasive SCC in four weeks. Inhibition or knockdown of YAP blocked OSCC onset *in vitro* and *in vivo*. Therefore, targeting YAP can be a possible avenue in mitigating tumour progression and metastasis in the context of OSCC. This could have marked implications for the routine treatment of OSCC providing future research can ascertain the safety, efficacy, and feasibility of VP therapy.

Despite the limited research on the applicability of YAP inhibitors to OSCC, the above-mentioned studies have significant implications for ongoing research and treatment of OSCC. In this regard, inhibitors of YAP, as well as TAZ, may provide a novel and effective option by exploiting multiple hits to cancer cells; optimising tumour regression, diminishing the resistance and enhancing sensitisation to various chemotherapy agents, reducing the time to relapse, and as neoadjuvant or adjuvant therapy to enhance responsiveness to systemic agents and radiotherapy/surgery [123,132,135].

## YAP Can be Regulated by DSG3, a Newly Identified Component in the Hippo Network

Cell-cell junctions, such as tight junctions and adherens junctions can stimulate Hippo signalling and transduce signals from the microenvironment to control the activity of the Hippo pathway [112]. However, little is known about the desmosomes and their components in the regulation of this pathway except for a few recent reports by us and another independent study [67,86,111,136,137]. Our study showed that DSG3 co-localises and forms a complex with phospho-YAP S127 (p-YAP) and sequesters it to the cell surface [67]. DSG3 silencing caused a reduction of YAP and p-YAP, accompanied by the downregulation of *YAP1* target genes, suggesting that DSG3 can affect YAP nuclear activity in addition to sequestering p-YAP to the cell surface [67]. In the reverse approach, YAP knockdown resulted in an increased expression of α-Catenin and DSG3, implying that analogous to α-Catenin, DSG3 can be negatively regulated by YAP [86,111]. These findings prompted us to propose a model that DSG3 may positively regulate YAP and YAP negatively modulated DSG3. However, our DSG3 gain-of-function study indicated that DSG3 overexpression induced p-YAP expression with an increased ratio of p-YAP/YAP suggesting that Dsg3 suppresses YAP activity by inducing p-YAP expression [86]. In support, a marked reduction of *YAP1* target genes, *CYR61/CTGF*, was seen in hDsg3.myc cells (DSG3 overexpression) compared to controls. Confocal microscopy confirmed a marked reduction of p-YAP nuclear expression coupled with enhanced cytoplasmic distribution in hDsg3.myc cells as opposed to control cells with predominantly p-YAP nuclear signals [86]. Functional analysis demonstrated that hDsg3.myc cells exhibited a marked reduction of cell migration in both the collective and colony settings. This finding phenocopies the YAP knockdown cells with reduced cell migration and concomitant upregulation of DSG3. The correlation between DSG3 and YAP activity was demonstrated in our two OSCC model systems (H157 displayed high DSG3 with low YAP transcriptional activity, whereas H413 had low DSG3 but high YAP transcriptional activity [86]). Therefore, the low DSG3 abundance coupled with high YAP activity is associated with enhanced cell migration or vice versa. Collectively, these lines of evidence suggest the model of mutual exclusive regulation between DSG3 and YAP activity and that DSG3 regulates YAP by inducing p-YAP expression and cytoplasmic relocation.

**Figure 3:**
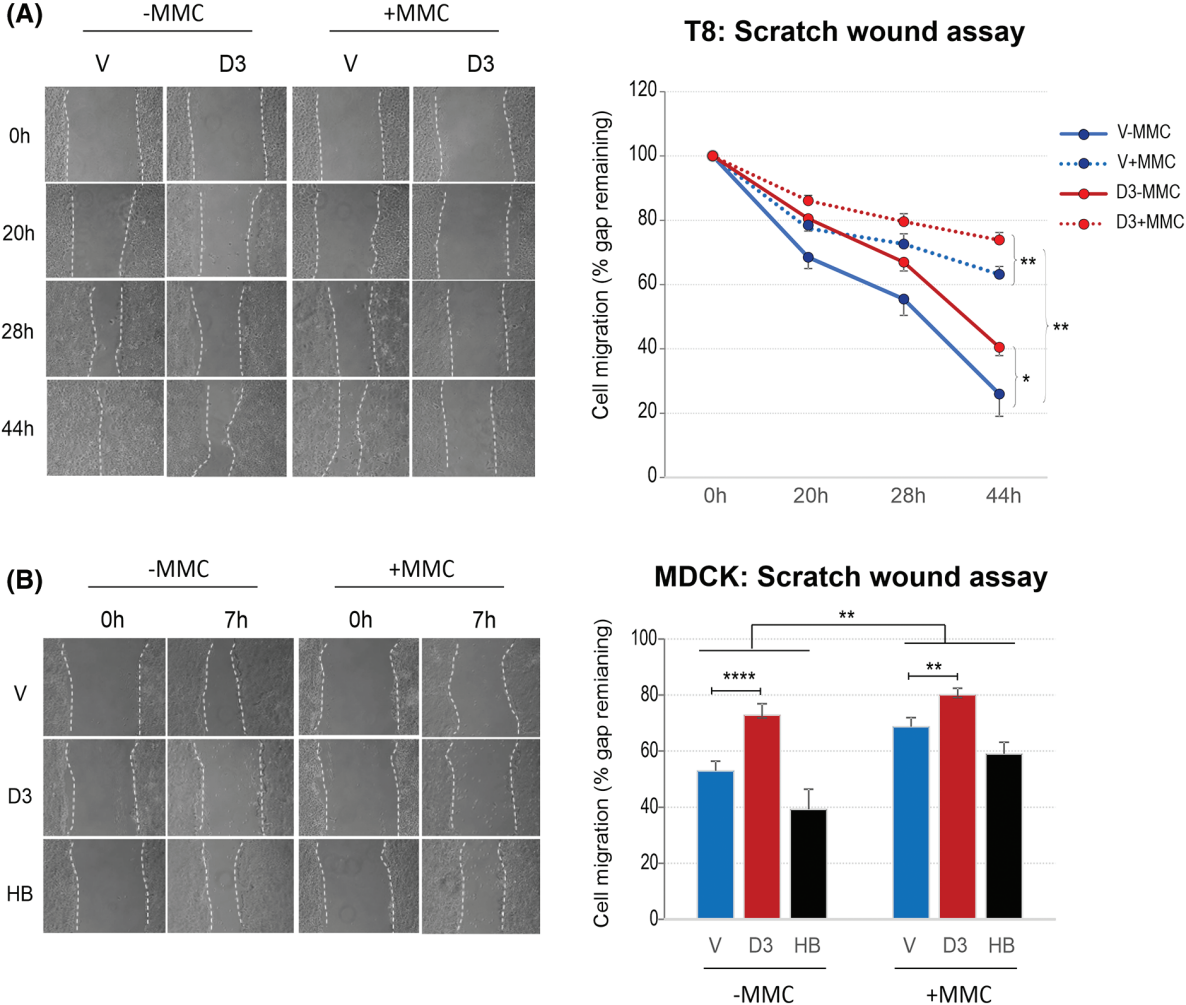
Overexpression of DSG3 suppresses cell migration capability. Scratch wound assay in two epithelial cell lines with or without DSG3 overexpression. (A) Cell migration of cutaneous carcinoma T8 keratinocyte lines over 44 h (n = 6, data pooled from two independent experiments, each in triplicates). (B) Cell migration of MDCK simple epithelial cells for 7 h (n = 9, data pooled from three independent experiments, each in triplicates). DSG3 overexpressing cells showed a slower migration rate than empty vector control lines in both cell types and DSG3 mutant expression restored cell migration activity to the baseline of MDCK control cells. MMC treated cells migrated significantly slower than the respective controls of untreated cells in both cell types (**p < 0.01). MMC, mitomycin C; V, empty vector control line; D3, DSG3 overexpression cell line; HB, DSG3 mutant containing EC1-4 domains and lacking the entire transmembrane and C-terminal domains (Mean ± SEM, *p*-values were determined by the Student’s *t-*test, *p < 0.05, **p < 0.01, ****p < 0.0001).

Recently, we expanded this study in additional epithelial cell lines (T8 and MDCK) in which DSG3 transduction and matched empty vector control lines were obtained [138,139] as well as an MDCK line with transduction of DSG3 mutant lacking the entire transmembrane domain and C-terminus [139] in order to establish a universal phenomenon of this pathway. Again, similar results in cell migration were obtained confirming that DSG3 overexpression suppresses cell migration and this was further corroborated in MDCK mutant cells that showed significant attenuation of p-YAP expression and associated restoration of cell migratory capability to the level of control cells (Fig. 3, data not shown). Therefore, we have established that DSG3 shares a common role with other cell-cell junction proteins to regulate the Hippo pathway that has an impact on cell migration, in this case via a mechanism of inducing p-YAP expression (and inhibiting YAP nuclear activity) and recruiting it to the cell surface to facilitate cell junction formation [136]. However, it remains unknown which Hippo kinases are involved in YAP phosphorylation that warrants further investigation.

To further investigate whether p-YAP is required for junction formation we inhibited YAP by VP that inhibits YAP-TEAD interaction. We found a marked reduction of YAP and p-YAP that caused a drastic effect on cell-cell junctions, leading to disintegration of both adherens junctions and desmosomes [136]. These findings suggest that YAP expression and the DSG3/p-YAP pathway are required for junction formation. Overexpression of DSG3 can rescue VP-mediated damage to cell junctions, resulting in better junction formation and stability [136]. We also showed that DSG3 overexpression *per se* was not necessary to enhance cell-cell adhesion demonstrated by the dispase assay that measures cell-cell adhesion strength [86]. Thus, our findings argue that DSG3 may function as a key upstream regulator in Hippo signalling to govern the contact inhibition of cell locomotion beyond its characterised role in desmosome adhesion.

**Figure 4:**
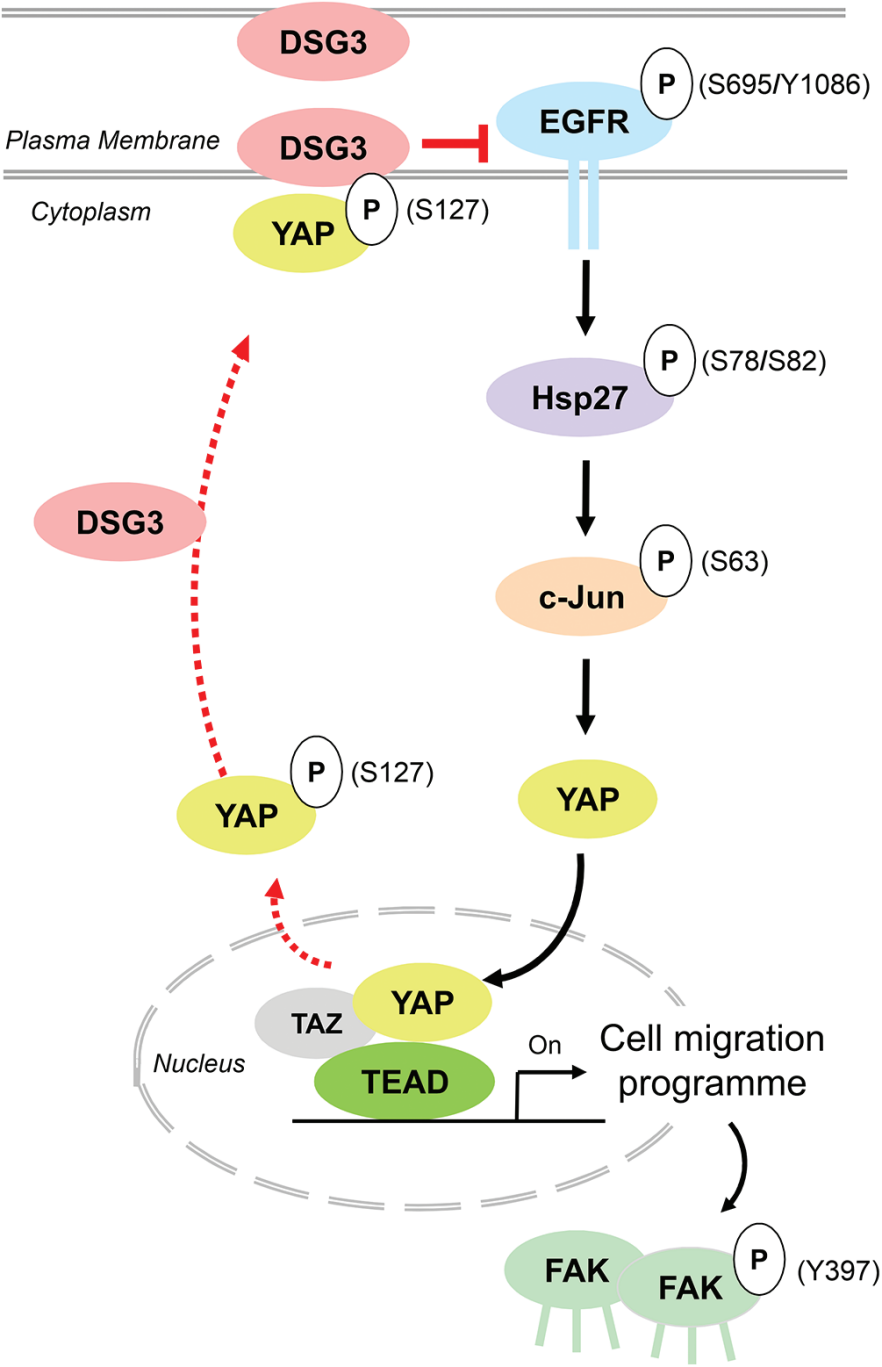
Schematic model that summarizes the major findings of Ahmad et al. (2022) [86]: DSG3 inhibits the EGFR-mediated YAP activation involved in OSCC cell migration. In the absence or low levels of DSG3 expression, YAP is activated through the EGFR/Hsp27/c-Jun signaling axis [140]. Phosphorylation of EGFR (S695 and Y1086) mediates downstream signaling events that phosphorylate and activate Hsp27 (S78/S82) and c-Jun (S63), leading to YAP nuclear translocation and binding to TEAD to promote the gene transcription program associated with cell migration that involves FAK and p-FAK Y397 [118,141]. However, this signaling process is abrogated by DSG3 expression, resulting in the suppression of cell motility in OSCC cells.

Mechanistically, we showed that overexpression of DSG3 suppressed EGFR signalling, including EGFR S695/Y1086 and its downstream heat shock protein 27 (Hsp27) S78/S82 and transcription factor AP-1 (c-Jun) S63 that trigger YAP activation. Thus, our study indicates that DSG3 inactivates YAP by impeding the EGFR/Hsp27/AP-1/YAP signalling axis in the control of collective migration of OSCC cells (Fig. 4). Consequently, overexpression of DSG3 rendered reduced FAK- and p-FAK expression and their mediated focal adhesion leading to inhibition of OSCC cell migration. In addition to DSG3, our study also suggested several desmosomal genes (*DSC1-3*, *DSG1/2*, *PKP1/2*, *JUP* and *DSP*) are likely involved in the negative regulation of YAP [86]. In summary, we have identified a mutually exclusive regulation between DSG3 and YAP nuclear activity. Furthermore, we have proven that DSG3 acts as an important upstream regulator of the Hippo-YAP pathway and suppresses the EGFR/Hsp27/AP-1/YAP signalling axis that has an impact on collective cell migration. Thus, our study reveals an unprecedented role of the DSG3/YAP pathway in OSCC. In an attempt to elucidate the pathogenesis of arrhythmogenic cardiomyopathy, another independent study has discovered that PKP2 deficiency-mediated modulation of intercalated discs in the heart is associated with the pathogenic activation of Hippo kinases. Thus this study highlights the vital role of PKP2 in the suppression of the Hippo pathway [137]. Our findings suggest that DSG3 acts as a pleiotropic protein depending on the cell context and the experimental setting [67,86,111,138,139,142]. Therefore, the regulation of Hippo by desmosomal components may largely be dependent upon the tissues and organs.

## Conclusions and Future Perspective

YAP overexpression is found in OSCC among other cancers. However, YAP abundance may not be indicative of its activity, but rather its predominantly nuclear localization. Thus, caution must be taken to draw a conclusion based on YAP abundance. Although YAP is required for efficient oral cancer cell migration, the heterogeneity of the Hippo-YAP pathway and partially overlapping activities of YAP/TAZ seem a common feature of OSCC cells and are likely contributing to resistance.

Importantly, we have uncovered a new paradigm that DSG3 and YAP activity exhibit mutually exclusive dependence and DSG3 acts as an important component in the Hippo network in the control of contact inhibition of cell locomotion. These findings pave the way for future research on the DSG3/YAP pathway in tumour cell biology and its implication in the diagnosis and treatment of OSCC. The experimental evidence supporting the role of YAP in the oncogenic process and emerging evidence for the therapeutic benefits of targeting this protein highlight an area of growing interest and research. Despite the recent advances in our understanding of the Hippo pathway in OSCC, there remain many unanswered questions. Further *in vivo* validation is absolutely necessary to corroborate our current *in vitro* findings. Additionally, the heterogeneity of YAP and TAZ activities in the control of cancer cell invasion and metastasis requires further in-depth study. It remains unclear whether p-YAP is necessary for junction formation although our initial findings suggest that this is the case [136]. A further study is needed in order to elucidate the role of the Hippo pathway in cell junction adhesion and structural integrity. We also observed that p-YAP existed in the nucleus of keratinocytes, however, its role in nuclear transcription activity remains undefined. Furthermore, we showed that DSG3 is able to regulate TAZ [136]. Our findings open a new avenue of research on these topics and the insight from these studies may help advance our understanding of various desmosomal diseases including both skin and heart conditions. It is worth noting that both DSG3 and YAP are identified as fitness genes and are found to be upregulated in cancers, including oral, lung and cutaneous SCC [142] (our unpublished data). Therefore, further studies will provide valuable information about the roles of these molecules in tumour cell biology and will have important implications for cancer progression and metastasis.

## Abbreviations

AMOT, angiomotin; CDKN2A, cyclin-dependent kinase inhibitor 2a; c-Jun, transcription factor AP-1; CMYC, cellular myelocytomatosis; COX-2, cyclo-oxygenase-2; CTGF, connective tissue growth factor; cTNM, clinical tumor-node-metastasis; CYR61, cysteine-rich angiogenic inducer 61; DNA, deoxyribonucleic acid; DSG3, desmoglein-3; ECM, extracellular matrix; EMT, epithelial to mesenchymal transition; EGFR, epidermal growth factor receptor; FAK, focal adhesion kinase; HNSCC, head and neck squamous cell carcinoma; HPV, human papillomavirus; Hsp27, heat shock protein 27; LATS1/2, large tumor suppressor 1 and 2 kinases; MDCK, Madin–Darby canine kidney; MMC, mitomycin C; MOB1A, MOB kinase activator 1A; MST1/2, mammalian Sterile 20-related 1 and 2 kinases; OSCC, oral squamous cell carcinoma; p-FAK, phosphorylated-focal adhesion kinase, Tyr379; PKP1, plakophilin-1; PKP3, plakophilin-3; PPARγ, peroxisome proliferator-activated receptor gamma; pTNM, histopathological tumor-node-metastasis; PTPN14, protein tyrosine phosphatase non-receptor type 14; p-YAP, phosphorylated yes-associated protein S127; RT-qPCR, quantitative reverse transcription polymerase chain reaction; SAV1, Salvador 1; SCC, squamous cell carcinoma; siRNA, small interfering ribonucleic acid; TAZ, transcriptional co-activator with PDZ-binding motif; TEAD, transcriptional enhanced associate domain; TNM, tumor-node-metastasis; UV, ultraviolet; VEGFR, vascular endothelial growth factor receptor; VGLL4, vestigial like family member 4; VP, Verteporfin; WHO, World Health Organization; YAP, yes-associated protein.

## Data Availability

No data has been submitted with this review article.
